# Protective Effect of Brazilian Propolis against Liver Damage with Cholestasis in Rats Treated with **α**-Naphthylisothiocyanate

**DOI:** 10.1155/2013/302720

**Published:** 2013-04-22

**Authors:** Tadashi Nakamura, Yoshiji Ohta, Koji Ohashi, Kumiko Ikeno, Rie Watanabe, Kenji Tokunaga, Nobuhiro Harada

**Affiliations:** ^1^Japan Beekeeping Co. Ltd., Gifu 500-8691, Japan; ^2^Department of Chemistry, Fujita Health University School of Medicine, Toyoake, Aichi 470-1192, Japan; ^3^Department of Clinical Biochemistry, Faculty of Medical Technology, Fujita Health University School of Health Sciences, Toyoake, Aichi 470-1192, Japan; ^4^Department of Clinical Medical Technology, Kagawa Prefectural College of Health Science, Mure-cho, Kagawa 761-0123, Japan; ^5^Department of Biochemistry, Fujita Health University School of Medicine, Toyoake, Aichi 470-1192, Japan

## Abstract

We examined the protective effect of Brazilian propolis against liver damage with cholestasis in rats treated with *α*-naphthylisothiocyanate (ANIT) in comparison with that of vitamin E (VE). Rats orally received Brazilian propolis ethanol extract (BPEE) (25, 50, or 100 mg/kg), VE (250 mg/kg) or vehicle at 12 h after intraperitoneal injection of ANIT (75 mg/kg) and were killed 24 h after the injection. Vehicle-treated rats showed liver cell damage and cholestasis, judging from the levels of serum marker enzymes and components. The vehicle group had increased serum total cholesterol, triglyceride, phospholipid, and lipid peroxide levels, increased hepatic lipid peroxide, reduced glutathione, and ascorbic acid levels and myeloperoxidase activity, and decreased hepatic superoxide dismutase activity. BPEE (50 mg/kg) administered to ANIT-treated rats prevented liver cell damage and cholestasis and attenuated these serum and hepatic biochemical changes except hepatic ascorbic acid, although administered BPEE (25 or 100 mg/kg) was less effective. VE administered to ANIT-treated rats prevented liver cell damage, but not cholestasis, and attenuated increased serum lipid peroxide level, increased hepatic lipid peroxide level and myeloperoxidase activity, and decreased hepatic superoxide dismutase activity. These results indicate that BPEE protects against ANIT-induced liver damage with cholestasis in rats more effectively than VE.

## 1. Introduction

Propolis (bee glue) is a resinous hive product collected by honeybee from various plant sources. Propolis has important pharmacological properties, and it can be used for a wide range of purposes as antiinflammatory, antioxidant, antibacterial, antiulcerous, and antitumor agents [1, 2]. Chemically, propolis obtained from different areas of the world is constituted by 50%–60% of resin, 30%–40% of wax, 5%–10% of essential oils, and 5% of pollen, besides microelements [3]. Propolis contains various organic compounds such as phenols, tannins, polysaccharides, terpenes, aromatic acids, and aldehydes [1, 3–6].

Propolis collected from different areas of the world has been reported to exert a protective effect against *in vivo* acute liver damage in rats treated with hepatotoxicants such as carbon tetrachloride [7–12], D-galactosamine [13, 14], acetaminophen [15, 16], and econazole [17] through its several pharmacological actions to suppress hepatic oxidative stress associated with lipid peroxidation [7–11, 13, 16, 17] to inhibit hepatic drug-metabolizing enzymes [11, 12, 14, 15] and to ameliorate hyperlipemia [7, 12]. However, it is still unknown whether propolis exerts a protective effect against *in vivo* acute liver damage with cholestasis in rats. 

A single treatment of experimental animals with *α*-naphthylisothiocyanate (ANIT) is known to induce liver damage with intrahepatic cholestasis [18–20]. This ANIT-induced liver damage with cholestasis is thought to be useful for studying the mechanisms of drug-induced cholestasis, because liver damage and cholestasis resulting from the administration of certain drugs (e.g., erythromycin estolate, chlorpromazine, and others) to humans are mimicked by ANIT administration to rats [20]. The mechanisms of ANIT-induced liver damage with cholestasis have been proposed but have not been entirely clarified yet. It has been suggested that hepatic reduced glutathione (GSH) contributes to the development of ANIT-induced liver damage with cholestasis by virtue of its ability to form a reversible S-conjugate of ANIT that is critical in shuttling ANIT into bile, where it is released in large and probably toxic concentrations [21]. It has also been suggested that inflammation mediated by infiltrated neutrophils contributes to the development of ANIT-induced liver damage with cholestasis in rats [21]. Furthermore, it has been shown in rats treated once with ANIT that lipid peroxidation induced by reactive oxygen species (ROS) generated via infiltrated neutrophils in the liver tissue is involved in the development of liver damage with cholestasis [22]. It has also been shown in ANIT-treated rats that disruption of hepatic antioxidant defense system contributes to the development of liver damage with cholestasis [23, 24]. 

 The ethanol extract of Brazilian propolis is known to possess antioxidant and anti-inflammatory properties [25–31]. Vitamin E (VE) is well known to exert antioxidant and anti-inflammatory actions [32–35]. Our resent report has shown that the ethanol extract of Brazilian propolis protects against stress-induced liver damage in rats, at least in part, through its antioxidant and anti-inflammatory actions as in the case of VE [36]. Furthermore, it has been shown that VE protects against liver cell damage, but not cholestasis, in rats with a single ANIT treatment, at least in part, through its antioxidant and anti-inflammatory actions [37]. 

We, therefore, examined the protective effect of the ethanol extract of Brazilian green propolis against ANIT-induced liver damage with cholestasis in rats in comparison with that of VE. 

## 2. Materials and Methods

### 2.1. Materials

ANIT, bovine serum albumin, 3,3′,5,5′-tetramethylbenzidine (TMB), *p*-coumaric acid, *RRR*-*α*-tocopherol (*α*-Toc) used for VE administration, superoxide dismutase (SOD) purified from bovine erythrocytes, and yeast glutathione reductase were purchased from Sigma (St. Louis, MO, USA); NADPH from Oriental Yeast Co. (Tokyo, Japan); chlorogenic acid from Tokyo Chemical Ind., Co. Ltd. (Tokyo, Japan); artepillin C, *L*-ascorbic acid, cinnamic acid, chrysin, *N*,*N*-dimethylformamide, *α*,*α*'-dipyridyl, 5,5′-dithiobis(2-nitrobenzoic acid) (DTNB), ethylenediaminetetraacetic acid (EDTA), Folin-Ciocalteu reagent, gallic acid, kaempferol, quercetin, reduced glutathione (GSH), 2-thiobarbituric acid, trichloroacetic acid (TCA), standard *α*-Toc and *δ*-tocopherol used for VE determination, Tween 80, and other chemicals from Wako Pure Chemical Ind., Ltd. (Osaka, Japan). All chemicals used were of reagent grade and were not further purified. 

### 2.2. Preparation of the Ethanol Extract of Brazilian Propolis

Brazilian green propolis was collected in the area of Minas Gerais in Brazil by MN Propolis Ind., Comércio e Exportacēo, Ltda (Mogi das Cruzes, SP, Brazil). The collected propolis (Lot no. KA-02) was provided by Japan Beekeeping Co. Ltd. (Gifu, Japan). The quality of the provided propolis had been certificated as follows: artepillin C, 10.1%; flavonoids, 41.1 mg/g; and bee wax, 5.6%. Ethanol extraction of Brazilian green propolis was conducted as follows: approximately 35 g of crude propolis was added to 100 mL of 95% ethanol and the mixture was kept at room temperature for 7 days. The final concentration of ethanol in the Brazilian propolis ethanol extract (BPEE) prepared was 80%. When the prepared BPEE was completely dried at 40°C, the content of solid components was estimated to be 13.2%. The content of flavonoids in BPEE was determined according to the method of Dowd [38]. 

### 2.3. Chemical Composition Analysis

The content of total flavonoid in BPEE is expressed as that of quercetin equivalents. The content of polyphenols in BPEE was determined by the Folin-Ciocalteau colorimetric method described in the report of Ahn et al. [39]. The content of total polyphenol in BPEE is expressed as that of gallic acid equivalents. The main constituents in BPEE were analyzed by HPLC according to the method described by Izuta et al. [27] except that the mobile phase consisting of 1% acetic acid in 55% methanol was replaced by the mobile phase consisting of 1% acetic acid in 69% methanol. The HPLC was performed on a reversed-phase Shim-Pack CLC-ODS (15 cm × 4.5 mm i.d., Shimadzu, Kyoto, Japan) column with water-methanol-acetic acid (30 : 70 : 1, v/v) as a mobile phase at a flow rate of 1 mL/min at 40°C. The volume of the BPEE sample injected to the column was 5 *μ*L. The detection of chlorogenic acid, *p*-coumaric acid, quercetin, cinnamic acid, kaempherol, chrysin, and artepllin C in BPEE was conducted at 290 nm and the content of each constituent was estimated using its authentic compound. 

### 2.4. Experimental Animals

Male Wistar rats aged six weeks were purchased from Nippon SLC Co. (Hamamatsu, Japan). The animals were housed in cages in a ventilated animal room with controlled temperature (23 ± 2°C) and relative humidity (55 ± 5%) with 12 h of light (7:00 to 19:00). The animals were maintained with free access to rat chow, Oriental MF (Oriental Yeast Co., Tokyo, Japan) and tap water for one week. All animals received humane care in compliance with the Guidelines of the Management of Laboratory Animals in Fujita Health University. This animal experiment protocol was approved by the Institutional Animal Care and Use Committee. 

### 2.5. Experimental Groups and Administrations of ANIT, BPEE, and VE

Rats were divided into 6 groups as follows: Control group: rats not given ANIT and either BPEE or VE (*n* = 5), ANIT group: rats treated with ANIT alone (*n* = 7), ANIT + BPEE(25) group: rats treated with ANIT and postadministered with BPEE at a dose of 25 mg/kg (*n* = 7), ANIT + BPEE(50) group: rats treated with ANIT and postadministered with BPEE at a dose of 50 mg/kg (*n* = 7), ANIT + BPEE(100) group: rats treated with ANIT and postadministered with BPEE at a dose of 100 mg/kg (*n* = 7), and ANIT + VE group: rats treated with ANIT and postadministered with VE (*n* = 7). ANIT treatment and postadministration of BPEE or VE were conducted as follows: ANIT was dissolved in olive oil. BPEE was diluted with 5% Tween 80 solution and the diluted BPEE contained 6% ethanol. Therefore, VE (*RRR*-*α*-Toc) was dissolved in 5% Tween 80 containing 6% ethanol (Tween 80-EtOH). In all groups with ANIT treatment, seven-week-old rats fasted for 15 h received an intraperitoneal (i.p.) injection of ANIT at a dose of 75 mg per kg body weight, that is, 1 mL of the ANIT solution (7.5 mg/mL) per 100 g body weight, as described previously [22–24, 37]. Age-matched rats fasted for 15 h in the control group received an i.p. injection of the same volume of olive oil used as vehicle. At 12 h after the initial i.p. injection of ANIT or vehicle (olive oil), rats in the groups with ANIT treatment were orally administered with BPEE at a dose of 25, 50, or 100 mg of solid components present in the extract per kg BW or the prepared VE at a dose of 250 mg per kg body weight. Namely, ANIT-treated rats received a single oral administration of 1 mL of the diluted BPEE solution, the VE solution, or vehicle (Tween 80-EtOH) per 100 g body weight. The dose of VE used in the present study was determined based on the data shown in our previous report [37]. One mL of Tween 80-EtOH used as vehicle per 100 g body weight was orally administered to rats in Control group at the same time point. All rats used were fasted and received water *ad libitum *during experiments.

### 2.6. Sample Preparation

At 24 h after the initial ANIT or vehicle injection, each rat was weighed and then was sacrificed under ether anesthesia at which time blood was collected from the inferior vena cava. Serum was separated from the collected blood by centrifugation. Immediately after sacrifice, each liver was perfused with ice-cold 0.9% NaCl to remove blood remaining in the tissue and then weighed after washing in an ice-cold 0.9% NaCl solution and wiping on a paper filter. The collected livers and serum were stored at −80°C until use. 

### 2.7. Assays of Serum Components and Enzymes

Serum alanine aminotransferase (ALT) and aspartate aminotransferase (AST) were assayed using a commercial kit of Transaminase II-Test Wako. Serum *γ*-glutamyl transpeptidase (*γ*-GTP) was assayed using a commercial kit of *γ*-GTP C-Test Wako. These enzyme activities are expressed as an international unit (IU/l). Serum total bilirubin and total bile acid were assayed using commercial kits of Bilirubin BII-Test Wako and Total bile acid-Test Wako, respectively. Total cholesterol (T-Chol), triglyceride, and phospholipid in serum were assayed using commercial kits of Cholesterol E-Test Wako, Triglyceride G-Test Wako, and Phospholipid C-Test Wako, respectively. These kits were obtained from Wako Pure Chemical Ind. Ltd. Co., Osaka, Japan. Lipid peroxide (LPO) in serum was fluorometrically measured by the thiobarbituric acid method of Yagi [40] using tetramethoxypropane as a standard. In this assay, the excitation and emission wavelengths were 515 and 553 nm, respectively. The amount of serum LPO is expressed as that of malondialdehyde (MDA) equivalents. 

### 2.8. Assays of Hepatic Components and Enzymes

The weight of each isolated liver was estimated using its relative weight (g/100 g body weight). The isolated liver tissue was homogenized in 9 volumes of ice-cold 50 mM Tris-HCl buffer (pH 7.4) containing 1 mM EDTA to prepare 10% homogenate using a Physcotron handy microhomogenizer (Microtec Co., Funabashi, Japan). The liver homogenate was used for the assays of GSH, *α*-Toc, ascorbic acid, and LPO. GSH in the liver homogenate was assayed by the DTNB method of Sedlak and Lindsay [41] using GSH as a standard. *α*-Toc in the liver homogenate was assayed by the HPLC method with electrochemical detection using *δ*-tocopherol as an internal standard as described in our previous report [42]. Ascorbic acid in the liver homogenate was assayed by the *α*,*α*'-dipyridyl method of Zannoni et al. [43]. The concentration of ascorbic acid was assayed using the standard curve of authentic *L*-ascorbic acid. LPO in the liver homogenate was spectrophotometrically assayed by the thiobarbituric acid method of Ohkawa et al. [44] using tetramethoxypropane as a standard except that 1 mM EDTA was added to the reaction mixtures. The amount of hepatic LPO is expressed as that of MDA equivalents. Hepatic SOD was assayed using a commercial kit of SOD Assay Kit-WST (Doindo, Kumamoto, Japan). Hepatic catalase and Se-glutathione peroxidase (Se-GSH-px) were assayed according to the methods of Bergmeyer [45] and Hochstein and Utley [46], respectively. Hepatic myeloperoxidase (MPO), an index of tissue neutrophil infiltration [47], was assayed by the method of Suzuki et al. [48]. For the assays of these enzymes, the isolated liver tissue was homogenized in 9 volumes of ice-cold 0.05 M Tris-HCl buffer (pH 7.4) using a Physcotron handy microhomogenizer. After sonication on ice for 20 sec using a Handy Sonic model UR-20P, the homogenate was centrifuged at 4°C (10,000 ×g, 20 min), and the resultant supernatant was dialyzed against 100 volumes of the same buffer at 4°C for 1 h using a microdialysis device (molecular weight cutoff 3,500, Bio-Tec International Inc., Belleuve, WA, USA). SOD activity in the dialyzed liver tissue supernatant was determined at 37°C using purified erythrocyte SOD (5,000 units/mg solid) as a standard. This activity is expressed as the unit (U) of authentic purified bovine erythrocyte SOD showing activity equivalent to the determined activity. Catalase activity in the dialyzed liver tissue supernatant was measured at 37°C by recording hydrogen peroxide (H_2_O_2_) decomposition at 240 nm in the reaction system (1.0 mL) consisting of 10 mM H_2_O_2_, an appropriate amount of the dialyzed liver supernatant, and 100 mM phosphate buffer (pH 7.0). One unit (U) of this activity is defined as the amount of enzyme decomposing 1 *μ*mol H_2_O_2_ as a substrate per min. Se-GSH-px activity in the dialyzed liver tissue supernatant was measured at 37°C by recording the decrease in absorbance at 340 nm following the oxidation of NADPH in the reaction system (1.0 mL) consisting of 2 mM GSH, 0.2 unit of yeast glutathione reductase, 1 mM NaN_3_, as a catalase inhibitor, an appropriate amount of the dialyzed liver supernatant, 2 mM H_2_O_2_ as a substrate, and 50 mM phosphate buffer (pH 7.0). One unit (U) of this activity is defined as the amount of enzyme oxidizing 1 *μ*mol NADPH per min. MPO activity in the dialyzed liver tissue supernatant was determined as follows: the dialyzed liver tissue supernatant was incubated at 60°C for 2 h to increase the recovery of MPO in liver tissues according to the method of Schierwagen et al. [49]. MPO activity in the heated liver tissue sample was assessed by measuring the hydrogen peroxide-dependent oxidation of TMB at 37°C. TMB was dissolved in* N*,*N*-dimethylformamide. One unit (U) of this enzyme is defined as the amount of enzyme causing a change in absorbance of 1.0 per min at 655 nm. Protein in the dialyzed liver tissue supernatant was measured using Protein Assay Rapid kit (Wako Pure Chemical Ind. Ltd. Co, Osaka, Japan). The amount of protein in the liver tissue supernatant is expressed as that of bovine serum albumin used as a standard. 

### 2.9. Histological Examination

Liver samples were taken from the central part of the right larger lobe of ANIT-treated rats with either BPEE or VE administration and untreated control rats at 24 h after ANIT treatment. They were fixed with 10% formalin in phosphate buffered saline for 24 h and then washed with tap water, dehydrated in alcohols, and embedded in paraffin. Sections 6-7 *μ*m thick were mounted in glass slides. Staining with hematoxylin and eosin (H-E) was performed in each slide and then histological examination was conducted under light microscopy. 

### 2.10. Statistical Analysis

All results obtained are expressed as means ± standard deviation (S.D.). The statistical analyses of the results were performed using a computerized statistical package (StatView). Each mean value was compared by one-way analysis of variance (ANOVA) and Bonferroni/Dunn for multiple comparisons. The significance level was set at *P* < 0.05. 

## 3. Results

### 3.1. Chemical Composition

The contents of total flavonoid and total polyphenol in BPEE used in the present study were 21.3 and 69.0 mg/g of solid propolis, respectively. The contents of *p*-coumaric acid, kaempferol, chrysin, and artepillin C in the extract were 14.9, 6.75, 2.38, and 47.8 mg/g of solid propolis, respectively. However, no chlorogenic acid, quercetin, and cinnamic acid were detected in the extract. 

### 3.2. Effects of BPEE and VE on f Liver Cell Damage and Cholestasis

**Figure 1:**
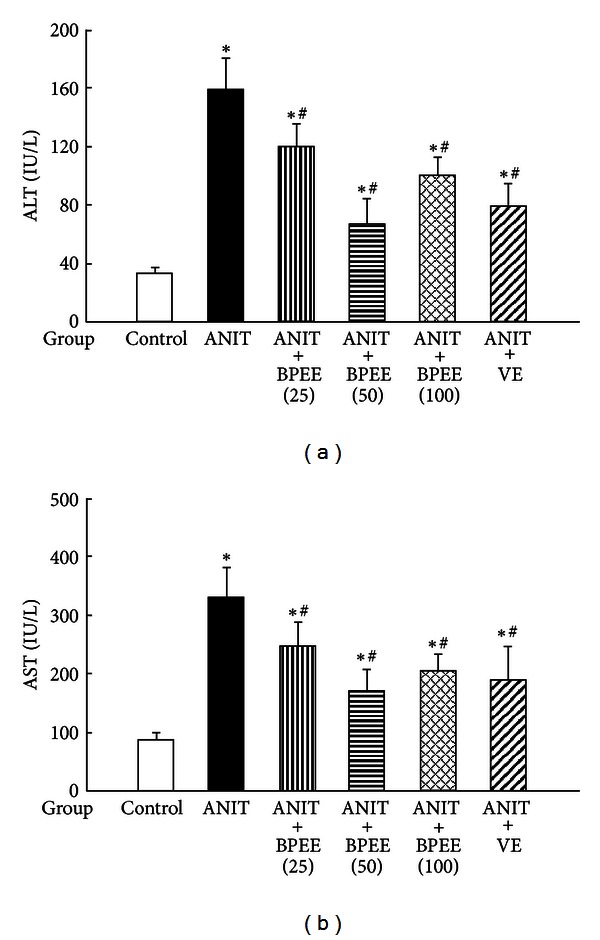
Effects of administered BPEE and VE on serum ALT (a) and AST (b) activities in ANIT-treated rats. Fasted rats in ANIT, ANIT + BPEE(25), ANIT + BPEE(50), ANIT + BPEE(100), and ANIT + VE groups were orally administered with vehicle (Tween 80-EtOH), 25 mg/kg of BPEE, 50 mg/kg of BPEE, 100 mg/kg of BPEE, and 250 mg/kg of VE, respectively, at 12 h after treatment with ANIT dissolved in olive oil (75 mg/kg, i.p.). Fasted rats in Control group were given olive oil and Tween 80-EtOH used as vehicle just before and at 12 h after ANIT treatment, respectively. ALT and AST in serum were assayed at 24 h after ANIT treatment as described in Section 2. Each value is a mean ± S.D. (*n* = 5 for Control group; *n* = 7 per each group for all groups with ANIT treatment). *P < 0.05 (versus Control group); ^#^ *P* < 0.05 (versus ANIT group).

**Figure 2:**
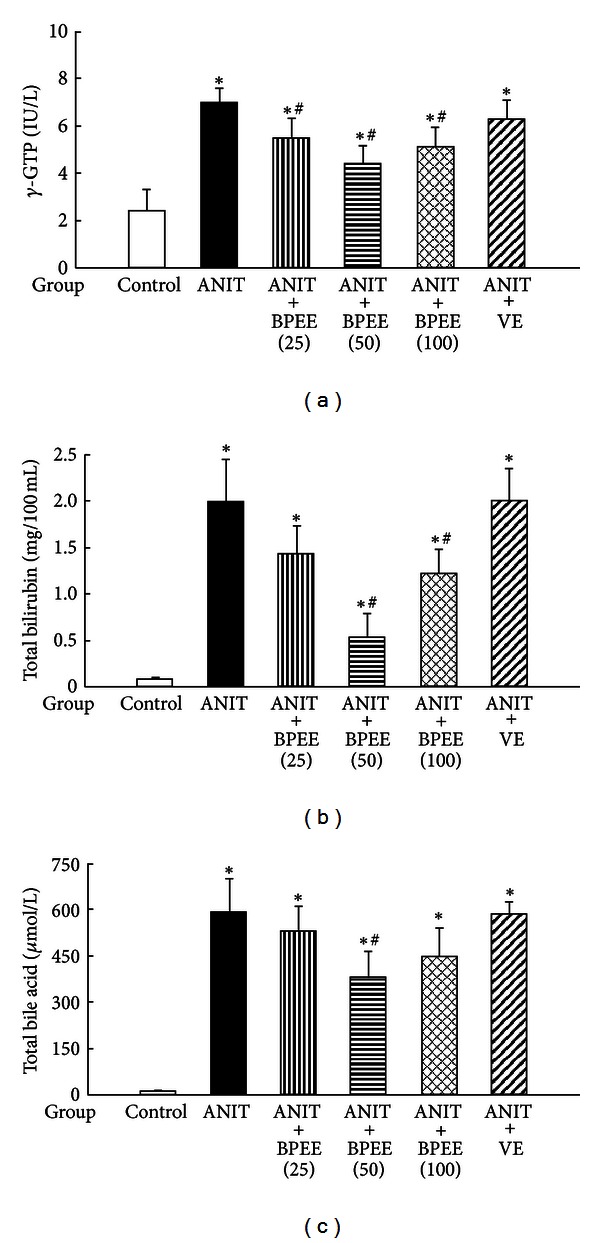
Effects of administered BPEE and VE on serum *γ*-GTP (a) activity and total bilirubin (b) and total bile acid (c) concentrations in ANIT-treated rats. Experimental condition and explanation are the same as described in the legend of Figure 1 except that *γ*-GTP, total bilirubin, and total bile acid in serum were assayed at 24 h after ANIT treatment as described in Section 2. Each value is a mean ± S.D. (*n* = 5 for Control group; *n* = 7 per each group for all groups with ANIT treatment). *P < 0.05 (versus Control group); ^#^ *P* < 0.05 (versus ANIT group).

Serum ALT and AST activities, indices of liver cell damage, and *γ*-GTP activity and total bilirubin and total bile acid concentrations, indices of biliary cell damage and cholestasis, were significantly higher in ANIT group than in Control group (Figures 1 and 2). The ANIT-induced increases in serum ALT, AST, and *γ*-GTP activities were significantly attenuated in ANIT + BPEE(25), ANIT + BPEE(50), and ANIT + BPEE(100) groups, while the ANIT-induced increases in serum ALT and AST activities were significantly attenuated in ANIT + VE group (Figures 1(a) and 2(a)). The attenuating effect on the ANIT-induced increase in serum ALT activity in ANIT + BPEE(50) group was significantly larger than that in ANIT + BPEE(25) and ANIT + BPEE(100) groups, while the attenuating effect on ANIT-induced increases in AST and *γ*-GTP activities in ANIT + BPEE(50) group tended to be larger than that in ANIT + BPEE25 and ANIT + BPEE(100) groups (Figures 1(a) and 2(a)). The attenuating effect on ANIT-induced increases in serum ALT and AST activities in ANIT + BPEE(50) group was almost equal to that in ANIT + VE group (Figure 1). The ANIT-induced increases in serum total bilirubin and total bile acid concentrations were significantly attenuated in ANIT + BPEE(50) group (Figures 2(b) and 2(c)). However, the ANIT-induced increases in serum total bilirubin and total bile acid concentrations were not attenuated in ANIT + BPEE(25) group (Figures 2(b) and 2(c)). Though the ANIT-induced increases in serum total bilirubin concentration were significantly attenuated in ANIT + BPEE(100) group, the attenuating effect in ANIT + BPEE(100) group was significantly less than that in ANIT + BPEE(50) group (*P* < 0.05) (Figures 2(b) and 2(c)). In ANIT + VE group, the ANIT-induced increases in serum total bilirubin and total bile acid concentrations were not attenuated at all (Figures 2(b) and 2(c)).

### 3.3. Effects of BPEE and VE on Live Histological Changes

**Figure 3:**
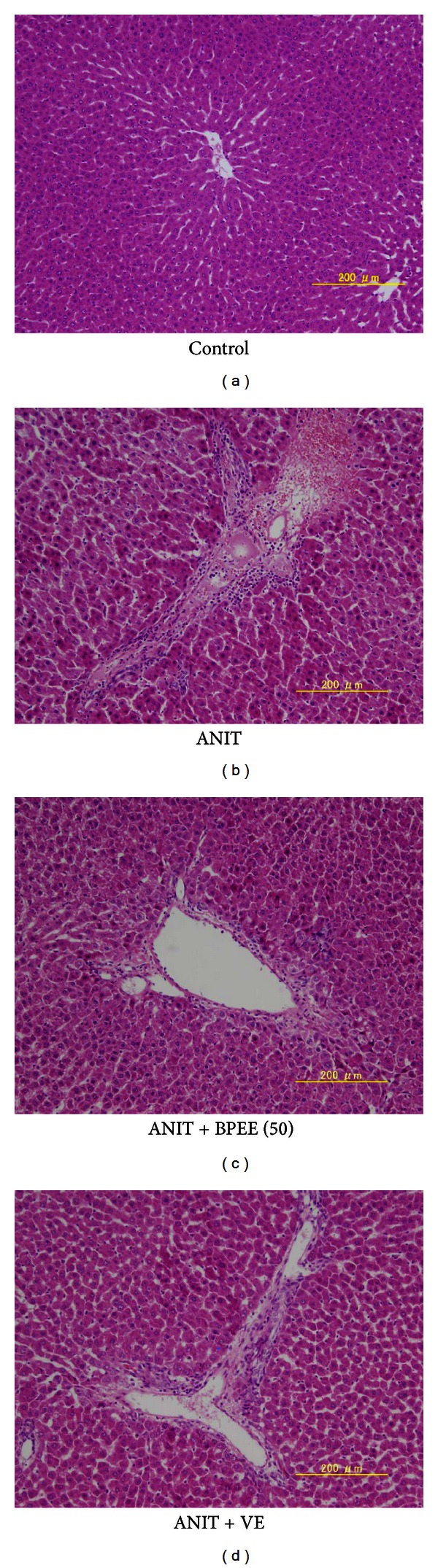
Histological Figures of liver cells from untreated control rats and ANIT-treated rats with and without either BPEE or VE. (a) Control group: little histological change was observed; (b) ANIT group: necrotic and degenerative changes with severe inflammatory cell infiltration were observed; (c) ANIT + BPEE50 group: dramatic decreases in necrotic and degenerative changes and inflammatory cell infiltration were seen; (d) ANIT + VE group: marked decreases in necrotic and degenerative changes and inflammatory cell infiltration were seen (H-E staining, original magnification ×100).

H-E-stained liver sections from Control group, ANIT group, ANIT + BPEE(50) group, and ANIT + VE group were examined for necrosis and inflammation. Control group showed little histological changes, ANIT group presented necrotic and degenerative changes with severe inflammatory cell infiltration, and ANIT + BPEE(50) and ANIT + VE groups presented clearly less necrotic and degenerative changes and less inflammatory cell infiltration (Figure 3). In addition, the histological change in ANIT + BPEE(50) group was similar to that in ANIT + VE group (Figures 3(c) and 3(d)). 

### 3.4. Effects of BPEE and VE on Serum Lipid Concentrations

**Figure 4:**
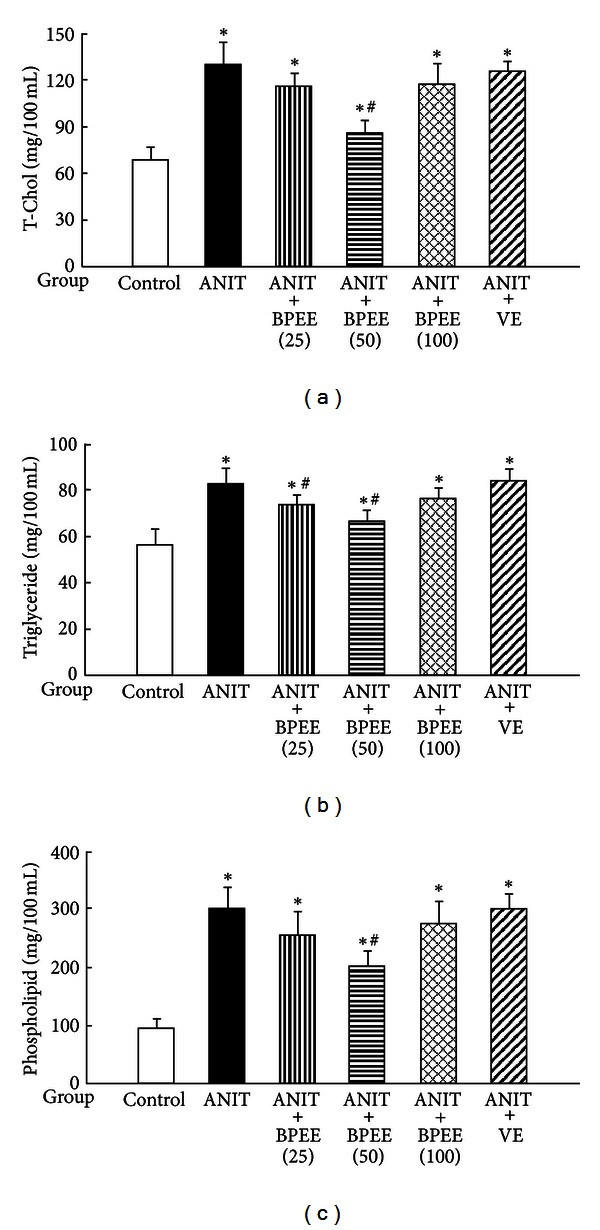
Effects of administered BPEE and VE on serum T-Chol (a), triglyceride (b), and phospholipid (c) concentrations in ANIT-treated rats. Experimental condition and explanation are the same as described in the legend of Figure 1 except that T-Chol, triglyceride, and phospholipid in serum were assayed at 24 h after ANIT treatment as described in Section 2. Each value is a mean ± S.D. (*n* = 5 for Control group; *n* = 7 per each group for all groups with ANIT treatment). *P < 0.05 (versus Control group); ^#^ *P* < 0.05 (versus ANIT group).

Serum T-Chol, triglyceride, and phospholipid concentrations were significantly higher in ANIT group than in Control group (Figure 4). In ANIT + BPEE50 group, the ANIT-induced increases in serum T-Chol, triglyceride, and phospholipid concentrations were significantly attenuated (Figure 4). However, only the ANIT-induced increase in serum triglyceride concentration was significantly attenuated in ANIT + BPEE(25) group, but no significant effects on the ANIT-induced increases in serum T-Chol, triglyceride, and phospholipid concentrations were observed in ANIT + BPEE(100) and ANIT + VE groups (Figure 4). 

### 3.5. Effects of BPEE and VE on Relative Liver Weight

**Figure 5:**
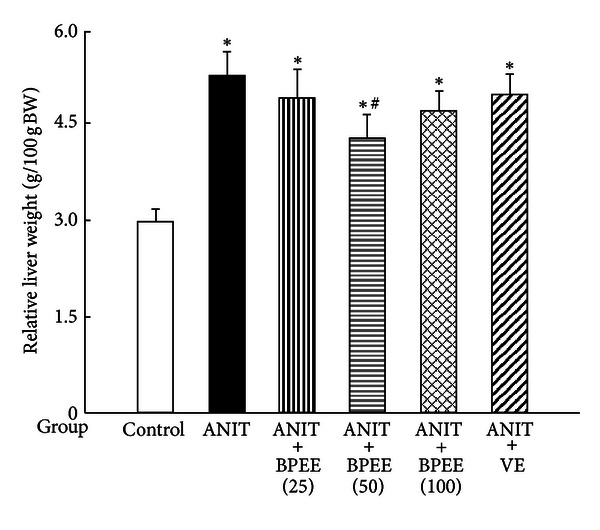
Effects of administered BPEE and VE on liver weight in ANIT-treated rats. Experimental condition and explanation are the same as described in the legend of Figure 1 except that the liver weight of each rat was estimated using its relative weight (g/100 g body weight) at 24 h after ANIT treatment as described in Section 2. Each value is a mean ± S.D. (*n* = 5 for Control group; *n* = 7 per each group for all groups with ANIT treatment). *P < 0.05 (versus Control group); ^#^ *P* < 0.05 (versus ANIT group).

ANIT group had significantly larger relative liver weight than Control group (Figure 5). In ANIT + BPEE(50) group, the ANIT-induced increase in relative liver weight was significantly attenuated, while no significant effect on the increase in the relative liver weight was observed in ANIT + BPEE(25), ANIT + BPEE(100), and ANIT + VE groups (Figure 5).

### 3.6. Effects of BPEE and VE on Serum and Hepatic LPO Concentrations

**Figure 6:**
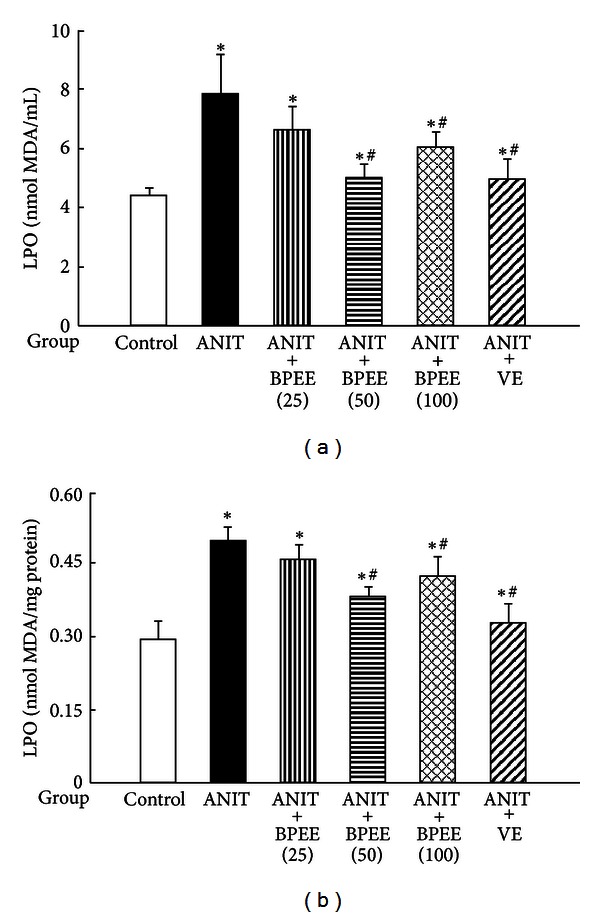
Effects of administered BPEE and VE on LPO concentrations in the serum (a) and liver (b) of ANIT-treated rats. Experimental condition and explanation are the same as described in the legend of Figure 1 except that LPO in serum and liver tissues was assayed at 24 h after ANIT treatment as described in Section 2. Each value is a mean ± S.D. (*n* = 5 for Control group; *n* = 7 for each group with ANIT treatment). *P < 0.05 (versus Control group); ^#^ *P* < 0.05 (versus ANIT group).

Serum and hepatic LPO concentrations were significantly higher in ANIT group than in Control group (Figure 6). The ANIT-induced increases in serum and hepatic LPO concentrations were significantly attenuated in ANIT + BPEE(50), ANIT + BPEE(100), and ANIT + VE groups, but no significant effect on both increases was found in ANIT + BPEE(25) group (Figure 6). The attenuating effects on the increases in serum and hepatic LPO concentrations in ANIT + BPEE(50), group were similar to those in ANIT + VE group, but were significantly larger than those in ANIT + BPEE(100) group (*P* < 0.05) (Figure 6). 

### 3.7. Effects of BPEE and VE on Hepatic Antioxidant Enzyme Activities

**Figure 7:**
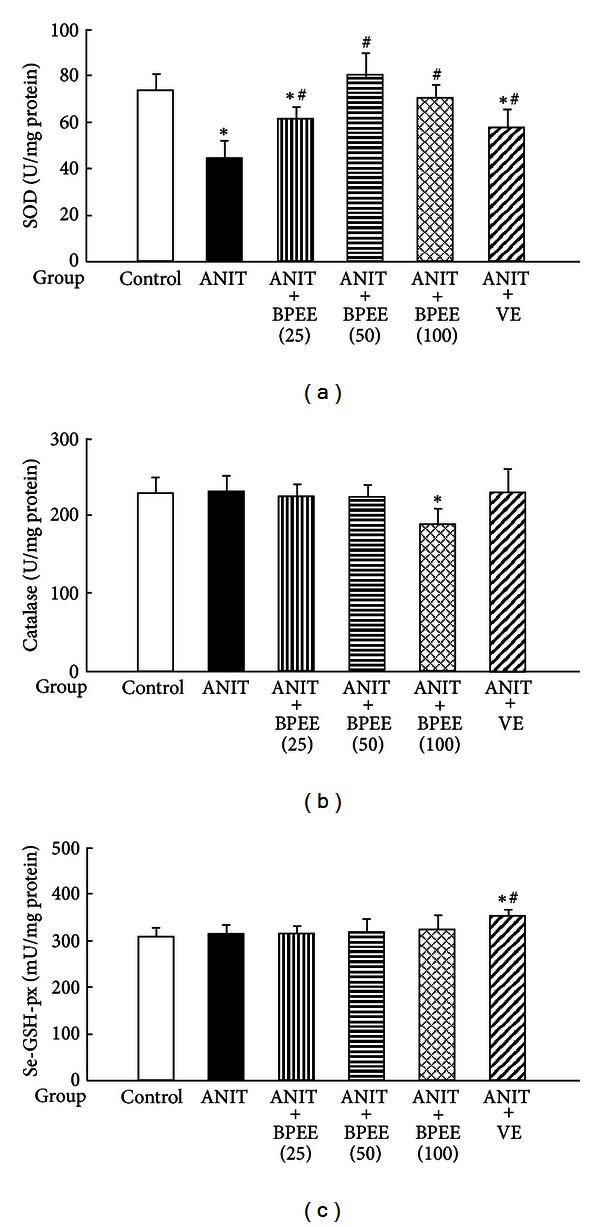
Effects of administered BPEE and VE on hepatic SOD (a), catalase (b), and Se-GSH-px (c) activities in ANIT-treated rats. Experimental condition and explanation are the same as described in the legend of Figure 1 except that SOD, catalase, and Se-GSH-px in liver tissues were assayed at 24 h after ANIT treatment as described in Section 2. Each value is a mean ± S.D. (*n* = 5 for Control group; *n* = 7 per each group for all groups with ANIT treatment). *P < 0.05 (versus Control group); ^#^ *P* < 0.05 (versus ANIT group).

Hepatic SOD activity was significantly lower in ANIT group than in Control group, although there were no significant differences in hepatic catalase and Se-GSH-px activities between both groups (Figure 7). The ANIT-induced decrease in hepatic SOD activity was significantly attenuated in ANIT + BPEE(25), ANIT + BPEE(50), ANIT + BPEE(100), and ANIT + VE groups, although ANIT + BPEE(50) group showed the most effective attenuation among the three ANIT + BPEE groups (Figure 7(a)). In addition, the hepatic SOD activity in ANIT + BPEE(50) or ANIT + BPEE(100) group was not different from that in control group (Figure 7(a)). ANIT + BPEE(25), ANIT + BPEE(50), and ANIT + VE groups had no significant effect on hepatic catalase activity but the enzyme activity was significantly reduced in ANIT + BPEE(100) group (Figure 7(b)). ANIT + BPEE(25), ANIT + BPEE(50) or ANIT + BPEE(100) group had no significant effect on hepatic Se-GSH-px activity but ANIT + VE group had a significant increase in that activity (Figure 7(c)). 

### 3.8. Effects of BPEE and VE on Hepatic Antioxidant Concentrations

**Figure 8:**
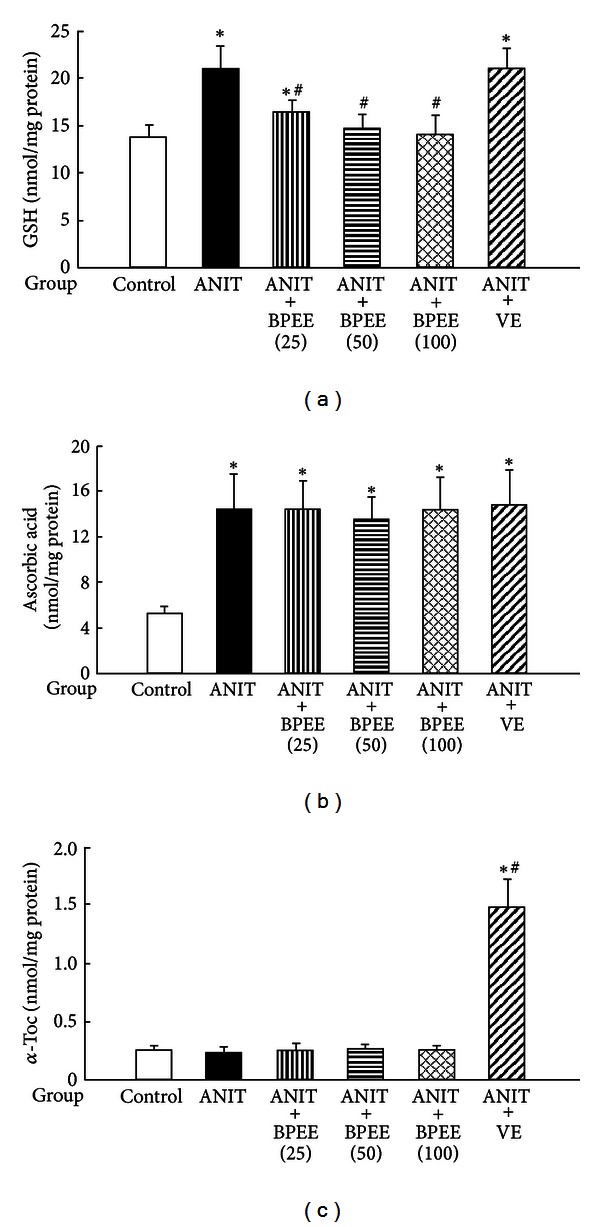
Effects of administered BPEE and VE on hepatic GSH (a), ascorbic acid (b), and *α*-Toc (c) concentrations in ANIT-treated rats. Experimental condition and explanation are the same as described in the legend of Figure 1 except that GSH, ascorbic acid, and *α*-Toc in liver tissues were assayed at 24 h after ANIT treatment as described in Section 2. Each value is a mean ± S.D. (*n* = 5 for Control group; *n* = 7 per each group for all groups with ANIT treatment). *P < 0.05 (versus Control group); ^#^ *P* < 0.05 (versus ANIT group).

Hepatic GSH and ascorbic acid concentrations were significantly higher in ANIT group than in Control group but there was no significant difference in hepatic *α*-Toc concentration between both groups (Figure 8). The ANIT-induced increase in hepatic GSH concentration was significantly attenuated in ANIT + BPEE(25), ANIT + BPEE(50) or ANIT + BPEE(100) group and the attenuating effect of BPEE occurred in a dose-dependent manner, while ANIT + VE group had no significant effect on the increased hepatic GSH concentration (Figure 8(a)). ANIT + BPEE(25), ANIT + BPEE(50), ANIT + BPEE(100), and ANIT + VE groups had no significant effect on the ANIT-induced increase in hepatic ascorbic acid concentration (Figure 8(b)). ANIT + BPEE(25), ANIT + BPEE(50), and ANIT + BPEE(100) groups showed no significant effect on the hepatic *α*-Toc concentration but ANIT + VE group had a significant and marked increase in the hepatic *α*-Toc concentration (Figure 8(c)). 

### 3.9. Effects of BPEE and VE on Neutrophil Infiltration

**Figure 9:**
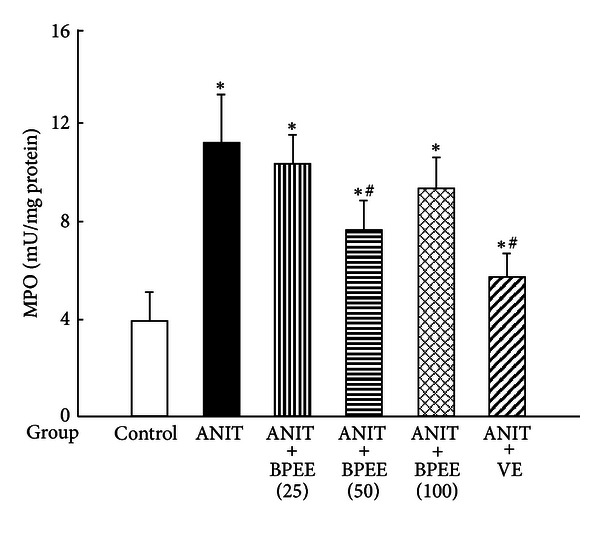
Effects of administered BPEE and VE on hepatic MPO activity in ANIT-treated rats. Experimental condition and explanation are the same as described in the legend of Figure 1 except that MPO in liver tissues was assayed at 24 h after ANIT treatment as described in Section 2. Each value is a mean ± S.D. (*n* = 5 for Control group; *n* = 7 for each group with ANIT treatment). *P < 0.05 (versus Control group); ^#^ *P* < 0.05 (versus ANIT group).

Hepatic MPO activity was significantly higher in ANIT group than in Control group (Figure 9). The ANIT-induced increase in hepatic MPO activity was significantly attenuated in ANIT + BPEE(50) and ANIT + VE groups, although neither ANIT + BPEE(25) group nor ANIT + BPEE(100) group had any significant effect on the increase in hepatic MPO activity (Figure 9). The attenuating effect on the ANIT-induced increase in hepatic MPO activity was significantly less in ANIT + BPEE(50) group than in ANIT + VE group (*P* < 0.05) (Figure 9). 

## 4. Discussion

The presence of *p*-coumaric acid, kaempferol, chrysin, and artepillin C in BPEE, which was prepared by extraction of Brazilian green propolis with 95% ethanol and used in the present study, was confirmed by the HPLC analysis used and the content of artepillin C was the highest among the contents of four constituents determined. This result was consistent with previous reports [5, 27, 50]. 

Our previous reports have shown that rats treated once with ANIT (75 mg/kg, i.p.) have liver cell damage and cholestasis, judging from the serum levels of ALT and AST, indices of liver cell damage, and *γ*-GTP, total bilirubin, and total bile acid, indices of biliary cell damage and cholestasis, at 24 h, but not at 12 h, after the treatment [22–24, 37]. In the present study, a single oral administration of BPEE or VE to ANIT-treated rats was conducted at 12 h after ANIT treatment, because this delayed administration of BPEE or VE is thought to be useful for providing the administration effect of the extract or the vitamin on ANIT-induced liver damage with cholestasis in a condition closer to the clinical situation. When liver cell damage was evaluated from the changes in serum ALT and AST activities, BPEE administered at a dose of 25, 50, or 100 mg/kg at 12 h after ANIT treatment was found to protect against ANIT-induced liver cell damage in rats. However, the protective effect of BPEE was higher at its dose of 50 mg/kg than at its dose of 25 or 100 mg/kg, indicating that the protective effect of BPEE against ANIT-induced liver cell damage is diminished at its high dose. VE (250 mg/kg) administered to ANIT-treated rats at 12 h after the treatment attenuated the increases in serum ALT and AST activities, as reported previously [37], and these effects were similar to those of BPEE (50 mg/kg). When the serum levels of *γ*-GTP, total bilirubin, and total bile acid were examined as indices of biliary damage and cholestasis, orally administered BPEE (25, 50, or 100 mg/kg) was found to attenuate the ANIT-induced increase in serum *γ*-GTP activity, although BPEE at a dose of 50 mg/kg was more effective than its dose of 25 or 100 mg/kg. In addition, BPEE administered at a dose of 50 mg/kg had a larger attenuating effect on the increases in serum total bilirubin and total bile acid concentrations than its dose of 25 or 100 mg/kg. The administered VE had no effect on the ANIT-induced increases in serum *γ*-GTP activity and total bilirubin and total bile acid concentrations, as reported previously [37]. Thus, orally administered BPEE (50 mg/kg) was found to protect against ANIT-induced liver damage with cholestasis in rats more effectively than the similarly administered VE (250 mg/kg). However, BPEE administered at a dose of 100 mg/kg was found to reduce its protective effect against ANIT-induced liver damage with cholestasis. Our recent report [36] has shown that a single oral preadministration of BPEE (50 mg/kg) to rats subjected to water-immersion restraint stress is more effective in protecting against liver oxidative damage induced by the stress than that of BPEE (100 mg/kg). In addition, orally administered BPEE (50 mg/kg) was found to attenuate histological changes associated with necrosis and inflammation in liver cells observed in rats treated with ANIT alone as in the case of orally administered VE. 

It is known that, in rats treated once with ANIT, increases in serum or plasma free cholesterol, cholesteryl ester, triglyceride, and phospholipid concentrations due to serum or plasma lipoprotein abnormality occur with the development of liver damage with cholestasis [51–53]. In the present study, ANIT-treated rats had increased serum T-Chol, triglyceride, and phospholipid concentrations at 24 h after the treatment and especially the treated rats had a marked increase in serum phospholipid concentration, as reported previously [51–53]. A single oral administration of BPEE (50 mg/kg) caused a significant attenuation of the ANIT-induced increases in serum T-Chol, triglyceride, and phospholipid concentrations, although the similarly administered BPEE (25 or 100 mg/kg) was less effective. By contrast, the similarly administered VE (250 mg/kg) had no significant effect on not only the increases in serum T-Chol and triglyceride concentrations, as reported previously [37], but also the increase in serum phospholipid concentration. Thus, orally administered BPEE (50 mg/kg) was found to be effective in attenuating increased serum T-Chol, triglyceride, and phospholipid concentrations in ANIT-treated rats. This attenuating effect of BPEE on ANIT-induced increases in serum lipid concentrations may be associated with amelioration of serum lipoprotein abnormality, although the attenuating mechanism is unclear at present. 

It has been shown in rats treated once with ANIT (75 mg/kg, i.p.) that the relative liver weight (g/100 g body weight) begins to increase before the appearance of liver damage with cholestasis and further increases after the appearance of liver damage with cholestasis [22, 54]. In the present study, a single oral administration of BPEE (50 mg/kg) to ANIT-treated rats at 12 h after the treatment caused a significant attenuation of the increase in relative liver weight found at 24 h, while the similarly administered BPEE (25 or 100 mg/kg) and VE (250 mg/kg) had no significant effect on the increased relative liver weight. Thus, orally administered BPEE (50 mg/kg) was found to suppress hepatic hypertrophy in ANIT-treated rats. However, both the mechanism for ANIT-induced hepatic hypertrophy in rats and the mechanism by which BPEE suppresses hepatic hypertrophy in ANIT-treated rats are unclear at present. 

The ethanol extract of Brazilian propolis is known to exert antioxidant action by scavenging ROS and by inhibiting lipid peroxidation [25–28]. VE is known to function as a scavenger of ROS and as a chain breaker for lipid peroxidation [32]. It has been shown that hepatic lipid peroxidation contributes to the development of liver damage with cholestasis in rats treated once with ANIT [22, 23, 37, 54]. It is known that rats treated once with ANIT (75 mg/kg, i.p.) have significant increases in hepatic and serum LPO concentrations at 24 h after the treatment, although a significant increase in LPO concentration in the liver, but not in the serum, of ANIT-treated rats occurs at 12 h after the treatment [22, 23, 37, 55]. In the present study, a single oral administration of BPEE (50 or 100 mg/kg) to ANIT-treated rats at 12 h after the treatment caused a significant attenuation of the increased hepatic and serum LPO concentrations found at 24 h, although BPEE at a dose of 50 mg/kg was more effective than its dose of 100 mg/kg. The similarly administered VE (250 mg/kg) caused a significant attenuation of the increased hepatic and serum LPO concentrations found at 24 h after ANIT treatment, as reported previously [37]. The ability of BPEE (50 mg/kg) to attenuate the ANIT-induced increases in hepatic and serum LPO concentrations was almost equal to that of VE (250 mg/kg). Therefore, it was suggested that BPEE orally administered to ANIT-treated rats could suppress lipid peroxidation occurring in the liver tissue through its antioxidant property. 

 It has been shown in rats treated once with ANIT (75 mg/kg, i.p.) that the hepatic SOD activity decreases at 24 h, but not 12 h, after the treatment, while the hepatic catalase and Se-GSH-px activities increase at 12 h, although the increased catalase and Se-GSH-px activities are returned to the levels of untreated control rats at 24 h [23, 24]. In the present study, a single oral administration of BPEE (25, 50, or 100 mg/kg) to ANIT-treated at 12 h after ANIT treatment caused a significant attenuation of the decreased hepatic SOD activity found at 24 h but the administered BPEE (50 mg/kg) exerted the highest effect and caused a complete return of the decreased hepatic SOD activity to the level of untreated control rats. The administered BPEE (25 or 50 mg/kg) had no effect on the hepatic catalase activity found at 24 h after ANIT treatment, while the administered BPEE (100 mg/kg) caused a significant reduction of the hepatic catalase activity. All doses of BPEE had no effect on the hepatic Se-GSH-px activity found at 24 h after ANIT treatment. By contrast, VE (250 mg/kg) administered to ANIT-treated rats did not affect the hepatic catalase activity but caused a significant increase in the hepatic Se-GSH-px activity, although the administered VE partially attenuated the ANIT-induced decrease in hepatic SOD activity. Thus, orally administered BPEE was found to ameliorate disrupted hepatic enzymatic antioxidant defense system associated with SOD in ANIT-treated rats. 

It has been reported that rats treated once with ANIT (75 mg/kg, i.p.) have increased hepatic GSH concentration at 24 h after the treatment, increased ascorbic acid concentration at 12 and 24 h, and unchanged hepatic *α*-Toc concentration at 12 and 24 h [23, 24, 37]. In the present study, a single oral administration of BPEE (25, 50, or 100 mg/kg) to ANIT-treated rats at 12 h after the treatment caused a significant attenuation of the increased hepatic GSH concentration found at 24 h but had no effect on the increased hepatic ascorbic acid concentration and the hepatic *α*-Toc concentration found at 24 h. In addition, the hepatic GSH concentration in ANIT-treated rats with administration of BPEE (50 or 100 mg/kg) was not different from that in untreated control rats. The similarly administered VE had no effect on the increased hepatic GSH and ascorbic acid concentrations found at 24 h after ANIT treatment, although the administered VE caused a marked increase in the hepatic *α*-Toc concentration, as reported previously [37]. It has been suggested that hepatic GSH plays a causal or permissive role in ANIT-induced liver damage with cholestasis in rats through formation of a reversible GSH conjugate of ANIT in the liver cells and transport of the GSH conjugate of ANIT into the bile, where it dissociates to free ANIT and GSH [21]. Jean et al. [56] have reported that increases in bile GSH and ANIT concentrations occur before an increase in hepatic GSH concentration in rats orally treated with ANIT (100 mg/kg). Therefore, it seems unlikely that the reduction of increased hepatic GSH concentration found at 24 h after ANIT treatment in rats by BPEE administered at 12 h affects the formation of a reversible GSH conjugate of ANIT in the liver cells. We have observed that oral administration of BPEE (25, 50, or 100 mg/kg) to ANIT-treated rats increases the concentration of nonprotein-SH including GSH in the serum in a dose-dependent manner (unpublished data). Therefore, there is a possibility that BPEE administered to ANIT-treated rats enhances the secretion of GSH from the liver tissue into the bloodstream, resulting in a reduction of the increased GSH concentration in the liver tissue. 

The ethanol extract of Brazilian propolis is known to exert anti-inflammatory action by inhibiting neutrophil infiltration and the generation of ROS in activated neutrophils [29–31]. VE is known to function as an anti-inflammatory agent through inhibition of the generation of ROS in activated neutrophils and neutrophil infiltration [33–35]. It has been suggested that infiltrated neutrophils in the liver tissue of rats treated with ANIT play a critical role in the development of ANIT-induced liver injury with cholestasis [21, 22]. We have shown that neutrophil infiltration into the liver tissue of rats treated once with ANIT (75 mg/kg, i.p.) increases at 12 h after the treatment, that is, before the appearance of liver damage with cholestasis and further increases at 24 h [22, 37]. In the present study, a single oral administration of BPEE (50 mg/kg) to ANIT-treated rats at 12 h after the treatment caused a significant attenuation of the increase in hepatic MPO activity, an index of tissue neutrophil infiltration [47], found at 24 h, although the similarly administered BPEE (25 or 100 mg/kg) had no significant effect on the increase in hepatic MPO activity. The similarly administered VE (250 mg/kg) exerted a significant attenuating effect on the increase in hepatic MPO activity, as reported previously [37], although the administered VE was more effective than BPEE administered at a dose of 50 mg/kg. These results were well consistent with the above-described histological observation of liver cells. Thus, orally administered BPEE (50 mg/kg) was found to inhibit neutrophil infiltration into the liver tissue of ANIT-treated rats like the case of orally administered VE. It has been shown that activated neutrophils mediate lipid peroxidation through the production of ROS via NADPH oxidase in the cells [55]. It has also been shown that MPO mediates lipid peroxidation in the presence of hydrogen peroxide and halide ions [57]. Accordingly, these findings allow us to indicate that orally administered BPEE protects against oxidative damage associated with excessive ROS generations via infiltrated neutrophils in the liver tissue of rats treated with ANIT possibly through its antiinflammatory action as in the case of orally administered VE. We have observed that BPEE in concentrations of a few *μ*g/mL causes a strong inhibition of the activity of authentic MPO isolated from human neutrophils (unpublished data). Therefore, it seems likely that orally administered BPEE contributes to its protective effect against oxidative damage caused by infiltrated neutrophils in the liver of ANIT-treated rats through inhibition of the activity of MPO in the infiltrated neutrophils.

As to the above-described decrease in hepatic SOD activity in ANIT-treated rats, it has been reported that the decrease in SOD activity in the liver of rats treated once with ANIT (75 mg/kg, i.p.) is due to a decrease in the activity of Cu, Zn-SOD present in the cytosol of liver cells [24]. Cu,Zn-SOD is known to be inactivated by H_2_O_2_ *in vitro* [58, 59]. It is known that the ethanol extract of Brazilian green propolis and several compounds present in the extract can scavenge H_2_O_2_ *in vitro* [28]. As described above, it was observed that BPEE (50 mg/kg) administered to ANIT-treated rats inhibited infiltration of neutrophils to enable to produce H_2_O_2_ via activated NADPH oxidase into the liver tissue. Therefore, it can be thought that BPEE (50 mg/kg) orally administered to ANIT-treated rats attenuates the reduction of SOD activity in the liver tissue by inhibiting neutrophil infiltration into the tissue and/or by scavenging H_2_O_2_ produced by infiltrated neutrophils in the tissue.

Artepillin C was present as a main constituent in BPEE used in the present study. It has been reported that artepillin C exerts antioxidant action by scavenging ROS and by inhibiting lipid peroxidation [28, 60–62]. Therefore, it is suggested that the attenuating effect of administered BPEE on increased LPO concentration and decreased SOD activity in the liver of rats treated with ANIT could be mainly due to the antioxidant action of artepillin C present in the extract.

As to the main reason why BPEE administered at a dose of 100 mg/kg was less effective in protecting ANIT-induced liver damage with cholestasis than BPEE administered at a dose of 50 mg/kg, the following matters could be considered: BPEE (100 mg/kg) administered to ANIT-treated rats reduced hepatic catalase activity and was less effective in inhibiting neutrophil infiltration into the liver tissue than BPEE (50 mg/kg) administered to the treated rats. 

In conclusion, the results of the present study indicate that a single oral administration of BPEE to rats treated once with ANIT before the onset of apparent liver damage with cholestasis protects against liver damage with cholestasis, although this protective effect of BPEE diminishes at its high dose. The present results also suggest that the protective effect of BPEE against ANIT-induced liver damage with cholestasis could be due to the antioxidant, antiinflammatory, antihyperlipemic, and antihypertrophic actions of the extract. The orally administered BPEE (50 mg/kg) was found to be more effective in protecting against ANIT-induced liver damage with cholestasis than the similarly administered VE (250 mg/kg). However, further investigation is needed to clarify the exact mechanism underlining the protective effect of BPEE against liver damage with cholestasis in rats treated with ANIT.
